# Association analysis of gut microbiota-metabolites-neuroendocrine changes in male rats acute exposure to simulated altitude of 5500 m

**DOI:** 10.1038/s41598-023-35573-y

**Published:** 2023-06-07

**Authors:** Jianan Wang, Shiying Liu, Yalei Xie, Chengli Xu

**Affiliations:** 1 Institute of Basic Medical Sciences, Chinese Academy of Medical Sciences, School of Basic Medicine, Peking Union Medical College, Beijing, 100005 China; 2 Center of Environmental and Health Sciences, Chinese Academy of Medical Sciences, Beijing, 100005 China

**Keywords:** Microbiology, Endocrinology

## Abstract

Hyperactivation of hypothalamic–pituitary–adrenal (HPA) axis and hypothalamic–pituitary–thyroid (HPT) axis were found in acute high altitude challenge, but the role of gut microbiota and metabolites is unknown. We utilized adult male Sprague–Dawley rats at a simulated altitude of 5500 m for 3 days in a hypobaric-hypoxic chamber. ELISA and metabolomic analyses of serum and 16S rRNA and metabolomic analyses of fecal samples were then performed. Compared with the normoxic group, serum corticotropin-releasing hormone (CRH), adrenocorticotropic hormone (ACTH), corticosterone (CORT), and thyroxine (tT_4_) were increased in the hypoxia group, whereas thyrotropin-releasing hormone (TRH) was decreased. *Bacteroides*, *Lactobacillus,* *Parabacteroides,* *Butyricimonas,* *SMB53,* *Akkermansia,* *Phascolarctobacterium,* and *Aerococcus* were enriched in hypoxia group, whereas [*Prevotella*], *Prevotella,* *Kaistobacter,* *Salinibacterium,* and *Vogesella* were enriched in normoxic group. Metabolomic analysis indicated that acute hypoxia significantly affected fecal and serum lipid metabolism. In addition, we found five fecal metabolites may mediate the cross-talk between TRH, tT_4_, and CORT with [*Prevotella*], *Kaistobacter,* *Parabacteroides,* and *Aerococcus*, and 6 serum metabolites may mediate the effect of TRH and tT_4_ on [*Prevotella*] and *Kaistobacter* by causal mediation analysis. In conclusion, this study provides new evidence that key metabolites mediate the cross-talk between gut microbiota with HPA and HPT axis under acute hypobaric hypoxia challenge.

## Introduction

The rapid ascent of plain people to high-altitude above 2500 m usually experience acute mountain sickness (AMS), which occurs with some combination of symptoms including insomnia, fatigue, dizziness, anorexia, and nausea, with vomiting^1^. Gastrointestinal problems^2^ may affect gut microbiota in high-altitude-exposed populations. It has been found that *Prevotella* was enriched in the feces of Tibetans at high altitude (3600 m), whereas *Bacteroides* was enriched in the Han stool^3^. Tibetans living at 4800 m have a flora enriched in butyrate-producing bacteria^3^. Expedition members exposed to high-altitude above 5000 m had a significant decrease in gut probiotics such as *Bifidobacteria* in feces, and a significant increase in gut pathogenic bacteria^4^.

Neuroendocrine hyperactivity may be involved in the regulation of immune function, vascular stress, energy metabolism, emotion, and sleep^5,6^. Several studies have demonstrated changes in various hormone concentrations at high altitude, including increased norepinephrine and cortisol^7,8^, an acute increase in thyroid-stimulating hormone (TSH), thyroxine (tT_4_), free thyroxine (fT_4_), triiodothyronine (tT_3_), and free triiodothyronine (fT_3_)^9–11^, which may then gradually recover or decrease under chronic hypoxia exposure^10–12^. However, the mechanisms of HPA and HPT axis activation under acute hypobaric hypoxia (AHH) are still not well understood.

Intriguingly, our previous work showed that gut microbiota, HPA axis, and HPT axis hormones have changed significantly in rats at a simulated altitude of 5500 m, especially in the acute phase^13^. Mechanisms were still not completely elucidated though correlation has been found between hormones and gut microbiota by Spearman correlation analysis.

As a potential endocrine organ, gut microbiota produces metabolites with signaling functions or chemicals with hormonal properties, such as short-chain fatty acids (SCFAs), neurotransmitters, precursors of neuroactive compounds, bile acids, and choline metabolites, gastrointestinal hormones, and bacterial components^14,15^. Metabolites are secreted by bacteria into the intestinal lumen and transported to effector organs (e.g., brain) through the blood. In turn, bacteria in the gut may respond to the host's hormones, which affect microbiota homeostasis and metabolite production, potentially affecting the host's pathological state. For example, elevated norepinephrine stimulates the growth of non-pathogenic commensal *E.* *coli* and other gram-negative bacteria in the gut^16^. Alterations in gut microbial composition and gut permeability also affect the HPA axis and HPT axis hormones^17^. Such cross-talk between host and gut microbiota may be susceptible to environmental stress and plays an important role in hypoxia adaptation.

Altogether, it will provide novel insights into the interaction mechanism of gut microbiota-metabolite-neuroendocrine to explore the critical metabolites that mediate the correlation between gut microbes and host neuroendocrine hormones under AHH exposure. Therefore, we explored the interaction between gut microbes and host HPT axis and HPA axis hormones mediated by fecal and serum metabolites in male Sprague–Dawley rats at a simulated altitude of 5500 m for 3 days and provide medical protective data for high-altitude populations.

## Results

### Effect of simulated altitude at 5500 m on HPT Axis and HPA axis

**Figure 1:**
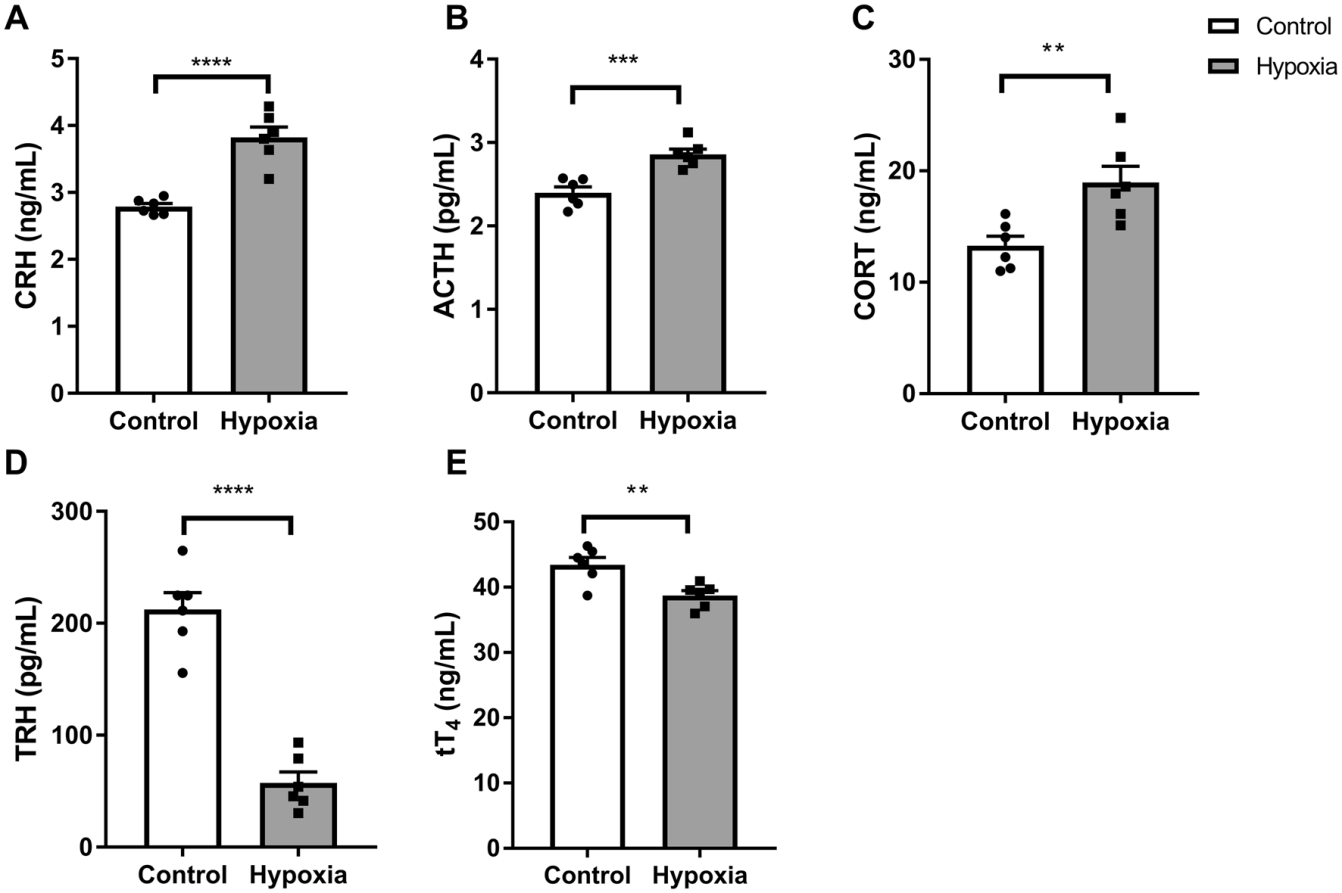
Effect of simulated altitude at 5500 m on HPT axis and HPA axis hormones. Serum levels of CRH (**a**), ACTH (**b**), CORT (**c**), TRH (**d**), tT_4_ (**e**). Data were expressed as mean ± SEM. n = 6/group. ∗∗*p* < 0.01, ∗∗∗*p* < 0.001, ∗∗∗∗*p* < 0.0001 compared to control group using two-tailed unpaired t-tests. *CRH* corticotropin-releasing hormone, *ACTH* adrenocorticotropic hormone, *CORT* corticosterone, *TRH* thyrotropin-releasing hormone, *tT*_*4*_ thyroxine.

Compared with the control, the body weight of hypoxia group was significantly reduced (control: 337.55 ± 21.74 g vs. hypoxia: 270.95 ± 9.88 g, t-test, *p* < 0.0001). Food intake of hypoxia group decreased during the acute hypoxia exposure (average food intake of hypoxia group: 9.65 g/day/rat, average food intake of control group: 25.29 g/day/rat). Compared with control, serum levels of CRH (t-test, *p* < 0.0001), ACTH (t-test, *p* = 0.0006), and CORT (t-test, *p* < 0.007) were significantly increased (Fig. 1a–c), whereas TRH (t-test, *p* < 0.0001) and tT_4_ (t-test, *p* = 0.0058) were significantly decreased (Fig. 1d, e) in hypoxia group. There were no significant changes in TSH, fT_4_, fT_3_, or tT_3_ levels (Supplementary Fig. 1a–d, t-test, *p* > 0.05).

### Effect of simulated altitude of 5500 m on gut microbiota

**Figure 2:**
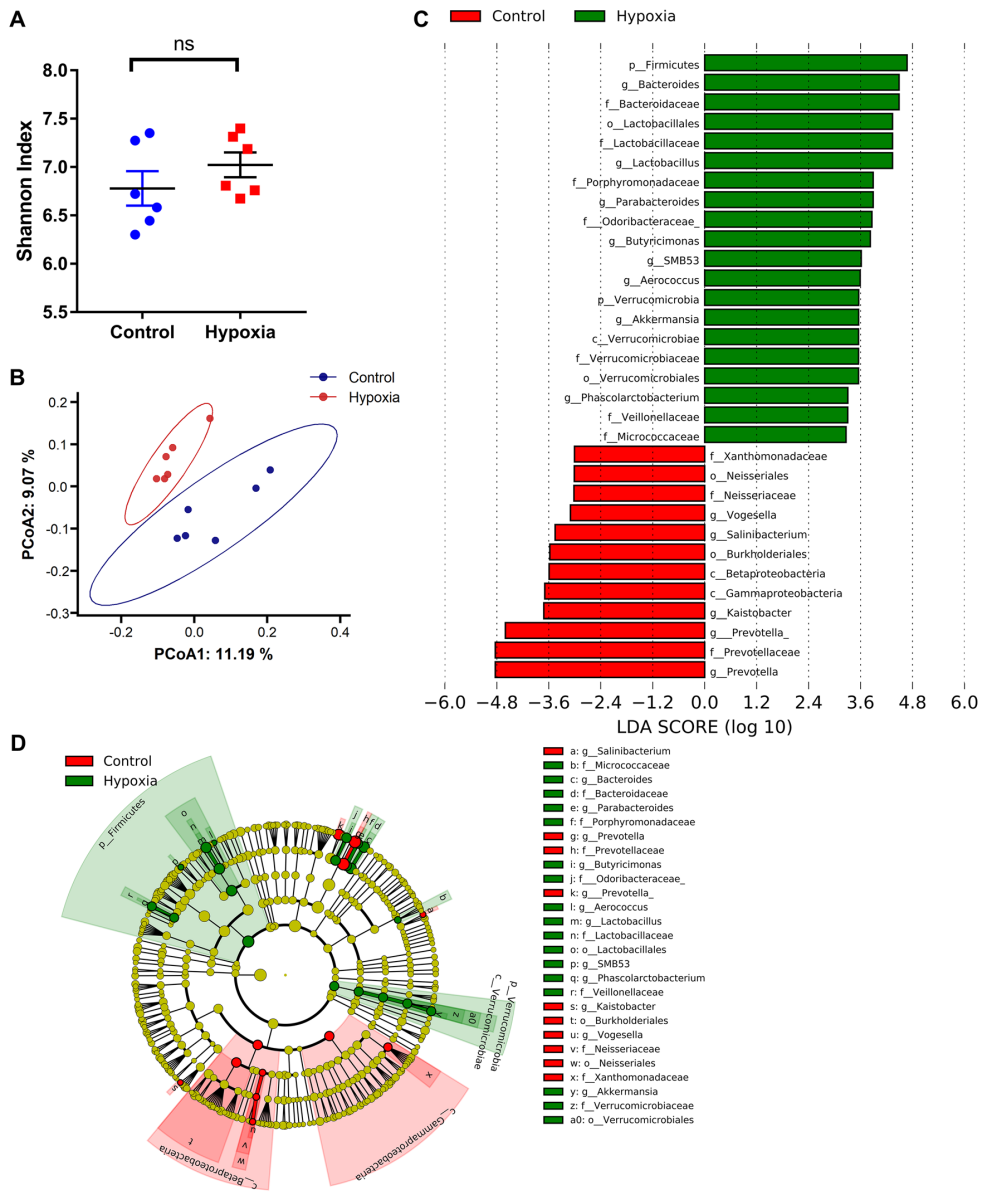
Effect of simulated altitude at 5500 m on gut microbiota. (**a**) Alpha diversity in gut microbiota was analyzed by Shannon Index using Mann Whitney test. Data were expressed as mean ± SEM, n = 6/group. (**b**) Principal coordinates analysis (Bray–Curtis distance) plot of the gut microbial community structure. (**c**) Histograms of ranked linear discriminant analysis (LDA) scores (threshold > 3, *p* < 0.05) computed for features differentially abundant between hypoxia group (green blocks) and control group (red blocks), and (**d**) cladogram mapping of the gut microbiota composition differences between hypoxia group (green blocks) and control group (red blocks) to taxonomic trees generated by LEfSe showed significant differences in gut microbial composition.

Based on 97% sequence similarity, 22,248 operational taxonomic units (OTUs) were identified and then assigned to 38 phyla, 99 classes, 160 orders, 198 families, and 254 genera. Acute hypoxia exposure had no significant influence on alpha diversity measured with Shannon index, Chao 1, or Observed OTUs (Fig. 2a, Mann Whitney test, *p* > 0.05). A Principal Co-ordinates Analysis (PCoA) plot of the Bray–Curtis distances confirmed that samples clustered and separated between groups (Fig. 2b). Linear discriminant analysis Effect Size (LEfSe)^18^ identified 13 differential genera. *Firmicutes* and *Verrucomicrobia* were significantly enriched in the hypoxia group at the phylum level (Fig. 2c, d, LDA score > 3, KW rank sum test and pairwise Wilcoxon test, *p* < 0.05). *Bacteroides*, *Lactobacillus*, *Parabacteroides*, *Butyricimonas*, *SMB53*, *Akkermansia*, *Phascolarctobacterium*, and *Aerococcus* were enriched in hypoxia group, whereas [*Prevotella*], *Prevotella,* *Kaistobacter*, *Salinibacterium,* and *Vogesella* were enriched in normoxic group at the genus level (Fig. 2c, d, LDA score > 3, KW rank sum test and pairwise Wilcoxon test, *p* < 0.05).

### Effect of simulated altitude at 5500 m on fecal and serum metabolomics

**Figure 3:**
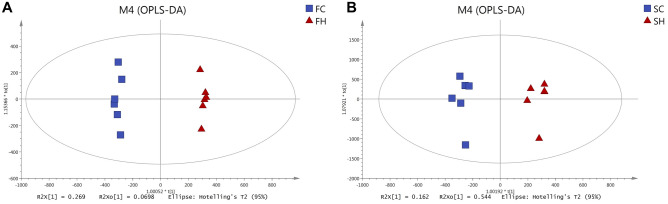
Effect of simulated altitude at 5500 m on fecal and serum metabolomics. Orthogonal partial least squares discriminant analysis (OPLS-DA) plot of fecal (**a**) and serum (**b**) metabolites. *FC* feces of control group, *FH* feces of hypoxia group, *SC* serum of control group, *SH* serum of hypoxia group. n = 6/group.

**Figure 4:**
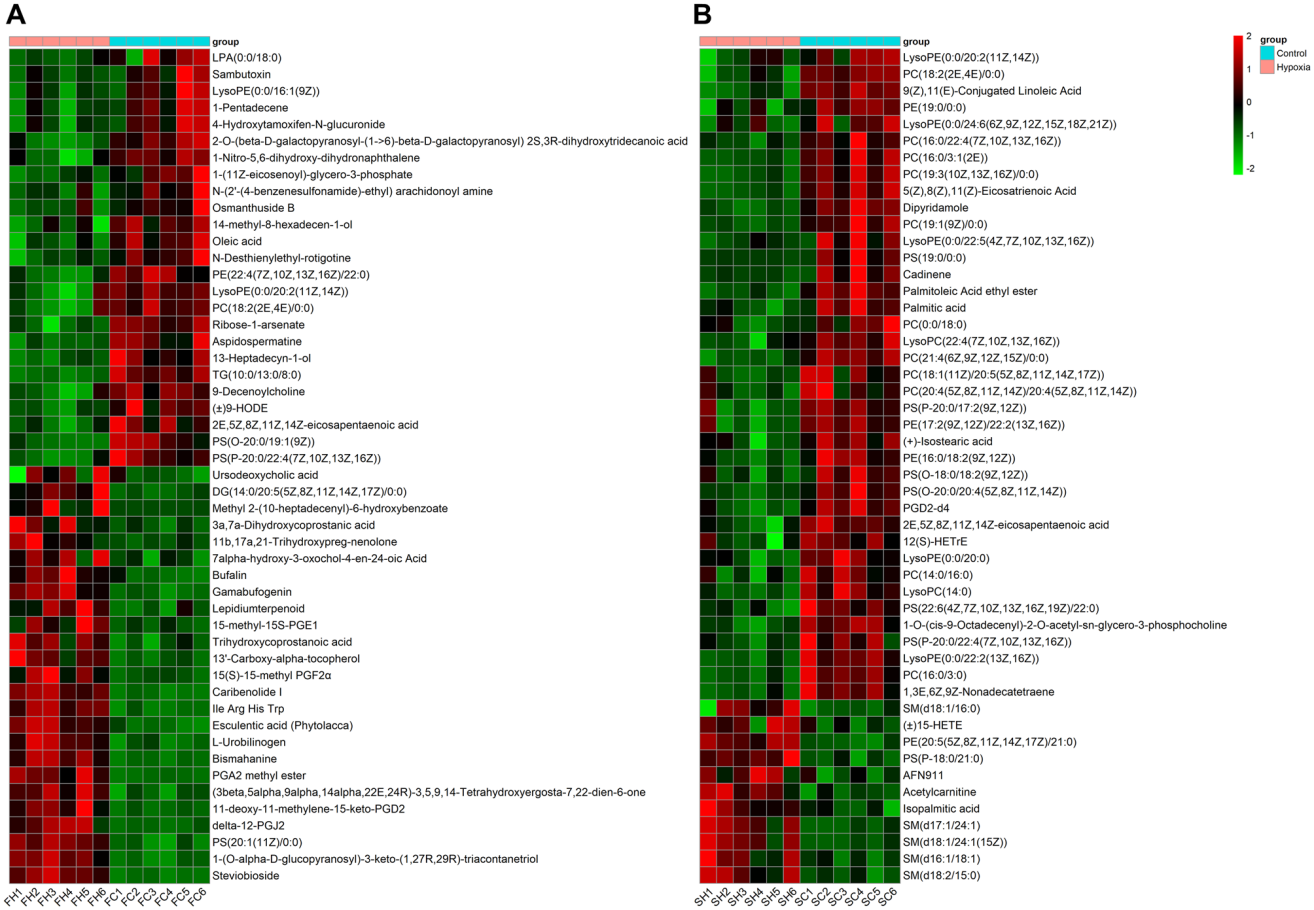
Heatmap of the top 50 altered metabolites ranked by Variable importance in the projection (VIP) in feces (**a**) and serum(**b**) with a cutoff value of VIP > 1 and *p* < 0.05 (t-tests).

**Figure 5:**
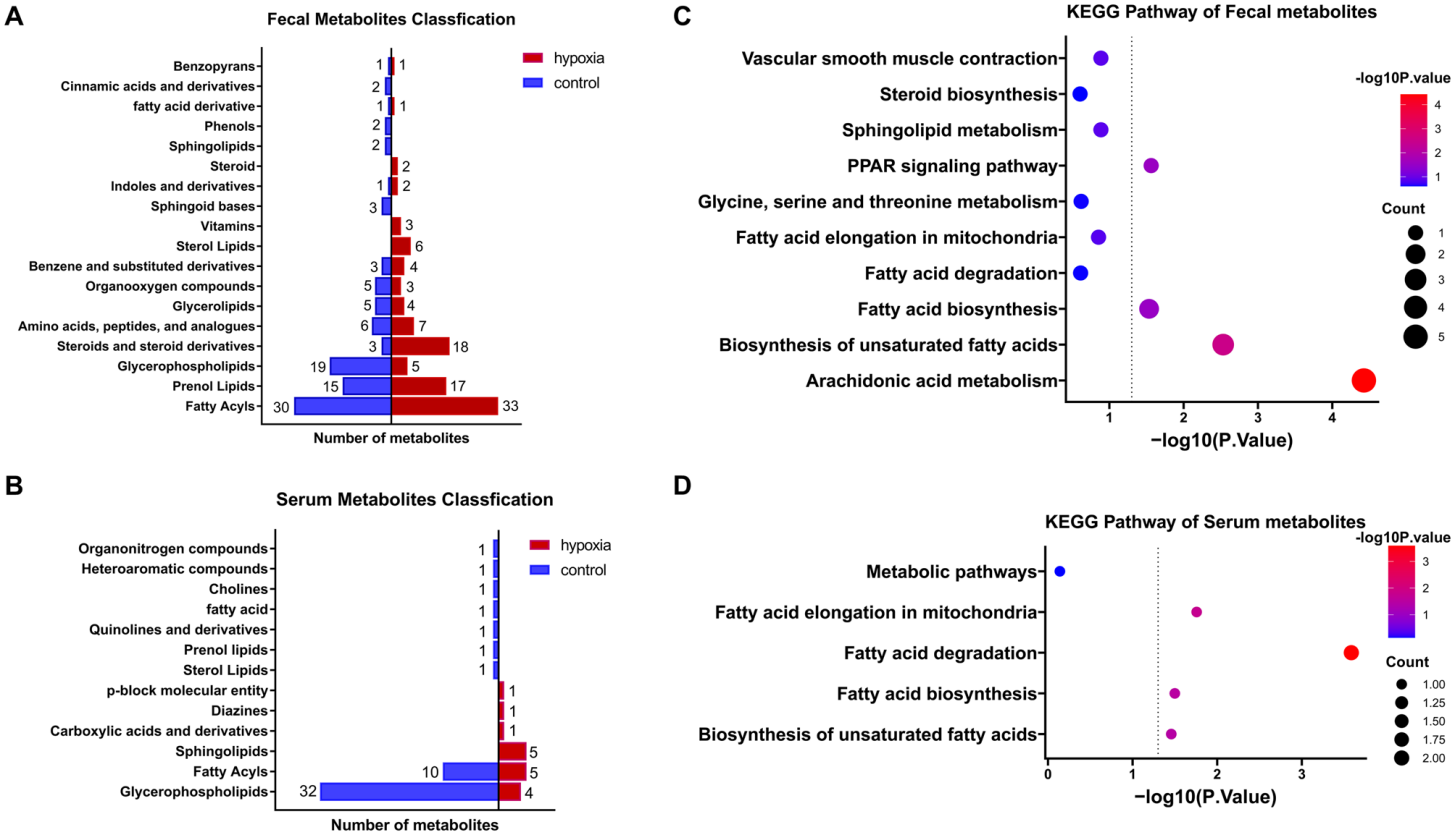
Classification and KEGG pathway analysis of altered metabolites. The butterfly chart showed the classification of altered metabolites (hypoxia vs. control) in feces (**a**) and serum (**b**). KEGG pathway analysis (https://www.kegg.jp/kegg/kegg1.html) of differential metabolites in feces (**c**) and serum (**d**). The bubble plot showed differential metabolites mapping to the corresponding metabolism pathways. The dotted lines indicated the cutoff *p* value of 0.05. The size of bubble indicates the count of altered metabolites included in each KEGG pathway. n = 6/group.

Differences in the metabolite profiles of feces and serum between hypoxia and normoxia groups were revealed by Orthogonal partial least squares discriminant analysis (OPLS-DA) (Fig. 3a, b). A total of 2945 metabolites were identified in feces and 1457 metabolites were identified in serum. Multivariate analysis identified 233 differential metabolites in feces and 66 differential metabolites in serum with cutoff value of variable importance in the projection (VIP) more than 1 and *p*-value less than 0.05 (t-tests, Supplementary Fig. 2a), of which 9 metabolites were co-downregulated in serum and feces, including five glyceryl phosphatides (LysoPE(0:0/20:2(11Z,14Z)), LysoPE(0:0/24:6(6Z,9Z,12Z,15Z,18Z,21Z)), PC(18:2(2E,4E)/0:0), PE(16:0/18:2(9Z,12Z)), and PS(P-20:0/22:4(7Z,10Z,13Z,16Z))), two long-chain fatty acids (2E,5Z,8Z,11Z,14Z-eicosapentaenoic acid and Palmitic acid), a prenol lipid (Cadinene), and a fatty acyls (Muricoreacin)(Supplementary Fig. 2b–j). Visualization of the top 50 differential metabolites ranked on VIP values in the feces and serum were shown in Fig. 4a, b, respectively. The differential metabolites in feces were mainly Fatty Acyls, Prenol Lipids, Glycerophospholipids, and Steroids and steroid derivatives (Fig. 5a). Significantly altered metabolites of serum mainly were Glycerophospholipids, Fatty Acyls, and Sphingolipids (Fig. 5b). Notably, most of the Glycerophospholipids and Fatty Acyls metabolites in serum were down-regulated under acute hypoxia exposure (Fig. 5b). Moreover, Kyoto Encyclopedia of Genes and Genomes (KEGG) pathway analysis was used to map altered serum and fecal metabolites to metabolism pathways. The result revealed that co-changed pathways in feces and serum were “Biosynthesis of unsaturated fatty acids” (Fig. 5c, d, fecal metabolites: *p* = 0.0029, serum metabolites: *p* = 0.0349) and “Fatty acid biosynthesis” (Fig. 5c, d, fecal metabolites: *p* = 0.0292, serum metabolites: *p* = 0.0317). In addition, changed pathways of fecal metabolites also included “Arachidonic acid metabolism” (Fig. 5c, *p* < 0.0001) and “PPAR signaling pathway”(Fig. 5c, *p* = 0.0274), and serum metabolites enriched in “Fatty acid degradation” (Fig. 5d, *p* = 0.0003) and “Fatty acid elongation in mitochondria” (Fig. 5d, *p* = 0.0175).

### Fecal and serum metabolites mediate the interaction between gut microbiota and host HPA axis and HPT axis hormones

**Figure 6:**
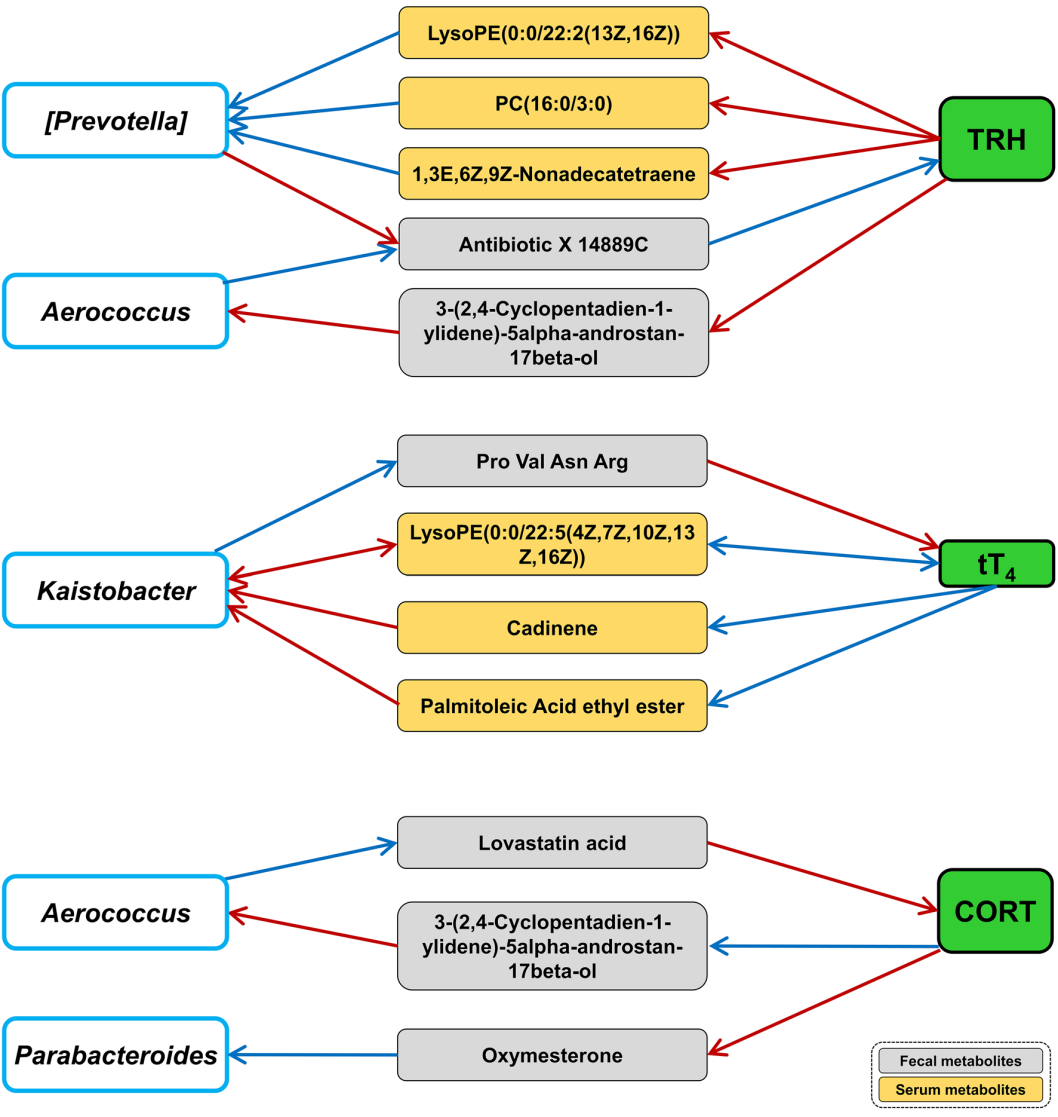
Causal mediation analysis revealed potential fecal and serum metabolites mediating the causal relationship between hormones with gut microbes. Blue lines, negatively coefficient; Red lines, positively coefficient. The arrows drown from metabolites to bacterial genus and hormones, and from bacterial genus and hormones to metabolites imply the conception that those indicators affect one another in the direction depicted. *CORT* corticosterone, *TRH* thyrotropin-releasing hormone, *tT*_*4*_ thyroxine.

Metabolites play an important role in the context of host-microbiota interactions. Hence, we then explored the key fecal and serum metabolites mediating the interaction between hormones with gut microbiota by causal mediation analysis (CMA). As shown in Fig. 6, a significant causal relationship could be identified between the gut microbiota and the altered hormones mediated by 6 serum metabolites and 5 fecal metabolites. The corresponding average causal mediation effect (ACME), average direct effect (ADE),and total effect can be found as Supplementary Table S1 online (*p* < 0.05).

Serum LysoPE(0:0/22:2(13Z,16Z)), PC(16:0/3:0), 1,3E,6Z,9Z-Nonadecatetraene could mediate the negative effect of TRH on [*Prevotella*] and fecal 3-(2,4-Cyclopentadien-1-ylidene)-5alpha-androstan-17beta-ol could mediate the positive effect of TRH on *Aerococcus*. Serum Cadinene, LysoPE(0:0/22:5(4Z,7Z,10Z,13Z,16Z)), and Palmitoleic Acid ethyl ester could mediate the negative influence of tT_4_ on *Kaistobacter*. Fecal Oxymesterone could mediate the negative effect of CORT on *Parabacteroides* and the negative influence of CORT on *Aerococcus* could be mediated by 3-(2,4-Cyclopentadien-1-ylidene)-5alpha-androstan-17beta-ol.

The negative influence of [*Prevotella*] and the positive influence of *Aerococcus* on TRH were mediated by the same fecal metabolite: antibiotic X 14889C. Fecal Pro Val Asn Arg could mediate the negative effect of *Kaistobacter* on tT_4_. fecal lovastatin acid (mevinolinic acid) could mediate the negative effect of *Aerococcus* on CORT.

## Discussion

Neuroendocrine response disturbance happened after exposure to high-altitude above 5000 m. Our study showed the HPA axis is significantly activated and TRH and tT_4_ of HPT axis were inhibited in male rats with acute exposure to high altitude, which is consistent with previous studies^7,19,20^. Neuroendocrine dysfunction associated with acute hypoxia stress may act as a trigger for decreased appetite^21^.

In addition, acute hypoxia exposure leads to changes in intestinal barrier permeability^22^ and gut microbiota composition^23^, resulting in host maladaption to high altitude and incidence of AMS^24^. Increased *Parabacteroides*, *Akkermansia*, *Lactobacillus*, *Bacteroides*, and decreased *Prevotella* were also found in human and animal models of AHH exposure^25–28^. *Bacteroides*, *Parabacteroides*, *Butyricimonas*, *Lactobacillus*, *Phascolarctobacterium*, and *Akkermansia* are intestinal commensal bacteria, most of whom are SCFAs producing bacteria^28–30^. Our results showed that they were enriched in hypoxia group. Previous study found higher *Prevotella* abundance has been shown to be involved in severe AMS symptoms^24^. *Prevotella* was involved in the regulation of metabolism^31^ and immunity^32^. Combined with the context, it may be suggested that adaptive changes in intestinal flora under acute hypoxia exposure may contribute to hypoxia adaptation.

Accumulating evidence points towards the interplay between the compositional and functional profile of the gut bacterial communities and neuroendocrine homeostasis^33–35^. Our study suggested TRH and tT_4_ may associate with *[Prevotella]* and *Kaistobacter* through serum lipid metabolites, including LysoPE(0:0/22:2(13Z,16Z)) (glycerophospholipids), PC(16:0/3:0) (glycerophospholipids), 1,3E,6Z,9Z-Nonadecatetraene (fatty acyls), Cadinene (prenol lipids), LysoPE(0:0/22:5(4Z,7Z,10Z,13Z,16Z)) (glycerophospholipids), and Palmitoleic Acid ethyl ester (fatty acyls). Previous study had found that *Prevotella* genus were related to peripheral thyroid homeostasis, increased in hyperthyroidism and decreased in hypothyroidism^35^. Pieces of evidence had shown the association between TRH and lipids^36,37^, and our finding indicated that TRH and tT_4_ correlated with serum lipids. Intestinal *Prevotella* spp., a highly abundant bacterial genus in the gut, may potentiates weight loss and decreases cholesterol levels^31^. *Prevotella_9* and *Prevotellaceae_NK3B31_group* were significantly positively correlated with glycerophospholipid metabolites^38^. LysoPE(0:0/22:5(4Z,7Z,10Z,13Z,16Z)) is a common Lysophospholipid (LPL), which may have a signaling transduction function^39^. Our study showed it may serve as a mediator to mediate bidirectional negative effect between tT_4_ with *Kaistobacter*. Those results may indicate that serum lipid metabolites may participate in lipid metabolism and act as a signaling molecule to mediate the effect of TRH on [*Prevotella*] and tT_4_ on *Kaistobacter*. In addition, HPT axis is possibly influenced by gut microbiota composition. Some studies have suggested that gut microbial composition may affect iodothyronine metabolism^40–42^. Whether and how microbial composition affect HPT axis is still a complex and redundant question. Although our study suggests gut microbe may affect TRH and tT_4_ through fecal metabolites, it shows few effect of acute hypoxia on the effector hormones in the HPT axis^40^. Changes in intestinal microbiota composition may affect peripheral T_4_ and T_3_ metabolism through microbe-derived metabolites, which limited the effect of inhibited TRH on peripheral T_4_ and T_3_ under acute hypoxia exposure^35,40,43^.

The disturbance of intestinal flora is closely linked to the activation of HPA axis. The communication between various gut microbiota disturbance and dysfunction of HPA axis is closely related to other systems, such as immunity, intestinal barrier and blood–brain barrier, microbial metabolites and intestinal hormones, etc^44^. Increased activity of HPA axis leads to increased intestinal barrier permeability and changes in gut microbiota composition, while the gut microbiota also leads to activation of HPA axis through microbe-derived metabolites and inflammation factors^45^. Our research suggests that most fecal metabolites mediate the correlation between *Aerococcus* with TRH and CORT. *Aerococcus*, a pathogen that may induce inflammation^46^, was significantly enriched in hypoxia group and associated with four fecal metabolites. Lovastatin acid, a highly effective hydroxymethylglutaryl-Coenzyme A reductase inhibitor^47^, is an inhibitor of cholesterol synthesis^48^. These results also further suggest that *Aerococcus* may interact with fecal steroid metabolites to participate in cortisol activation and regulation under hypoxia.

Besides, *Parabacteroides* were found to be influenced by CORT through the fecal metabolites Oxymesterone (Steroid). *Parabacteroides* are anti-inflammatory bacteria by producing acetate^30^, which is significantly elevated at acute hypoxia exposure. The abundance of *Parabacteroides* is significantly negatively correlated with serum CORT^49^. Several studies found that Oxymesterone, a steroid derivative, selectively suppressed 11β-hydroxy steroid dehydrogenase 2 (11β-HSD2) -dependent glucocorticoid inactivation and competitively bound to the cortisol oxidation site of 11β-HSD2^50^, leading to activation of the mineralocorticoid receptor (MR). Our results further suggested that *Parabacteroides* may be influenced by CORT through Oxymesterone under acute hypoxia exposure.

There are several limitations in this study. First, CMA analysis only suggests the target and direction of potential association between microbiota, metabolites, and neuroendocrine for the further study, rather than certified causal relationship, which still needs to be further verified by functional experiments. Second, the study only included acute phase of high-altitude exposure, concerning about the potential relationship of intestinal flora and neuroendocrine response of AMS. More additional research is needed for chronic hypoxia exposure. Third, as a preliminary exploratory study, the sample size used in this study is 6/group, and the conclusions are more speculative. Subsequent functional experimental studies are still needed for verification.

## Conclusion

In summary, the present study emphasized the dysfunction of HPA axis and HPT axis hormones, the disturbance of gut microbiota, and the altered fecal and serum metabolites in male Sprague–Dawley rats at a simulated altitude of 5500 m. Bidirectional association between gut microbes with TRH, tT_4_, and CORT through multiple serum and fecal metabolites may play a key role in hypoxia adaptation. However, this study explored the mediator metabolites of the interaction between neuroendocrine and intestinal microbiota through the combination of microbiome and metabolomics using causal mediation analysis, and a large number of studies are needed to further confirm the results of this study.

## Methods

### Rats

All rats experimental procedures were approved by the Animal Care and Use Committee of Chinese Academy of Medical Sciences & Peking Union Medical College (Approve No. ACUC-A02-2022-039) and conducted in accordance with guideline for ethical review of animal welfare (GB/T 35892-2018). Specific pathogen free (SPF) male Sprague–Dawley rats (Beijing Weitong Lihua Laboratory Animal Technology Co., Ltd.), 10-week-old, were housed in an animal room with 12:12 dark–light cycle, temperature of 20 ± 4 °C, and humidity of 30–60%. All efforts were made to minimize the number of animals used and their suffering. All experiments were carried out in compliance with the ARRIVE guidelines.

### Experiment protocols

After 1 week of pre-adaptation, rats were randomly separated into two groups. The hypoxia group (n = 6) was placed in a hypobaric oxygen chamber (FLYDWC50-IC) that mimics an altitude of 5500 m (379 mmHg), while the control group (n = 6) was kept in a normoxic environment (Beijing, China. 52 m, 760 mmHg). Rats received regular murine chow (300 g/d/cage, 3.44 kcal/g; 12.95% kcal from fat; Beijing Keao Xieli Feed Co., LTD.) and the chamber was opened for 20 min per day to add murine chow and water and record body weight and total food intake of each group. After 3 days of hypoxia and normoxia exposure, 3% sodium pentobarbital was used for deep anesthesia, and the rectal terminal stool was immediately taken from the abdomen, and immediately frozen at −80 °C. The left ventricular arterial blood sample was collected at 9:00 a.m. Serum was separated and stored at −80 °C until assayed.

### Serum hormone tests of HPA axis and HPT axis

Serum levels of thyrotropin-releasing hormone (TRH, CSB-E08040r), TSH (CSB-E05115r), tT_4_ (CSB-E05082r), fT_4_ (CSB-E05079r), tT_3_ (CSB-E05085r), fT_3_ (CSB-E05076r), corticotropin-releasing hormone (CRH, CSB-E08038r), adrenocorticotropic hormone (ACTH, CSB-E06875r), corticosterone (CORT, CSB-E07014r) were analyzed by ELISA kit from Cusabio Biotech Co, Ltd. All hormones were detected following the manufacturer’s guidelines.

### Gut microbiota analysis

Total DNA in fecal sample (250 mg) was extracted by QIAamp^®^ PowerFecal DNA Kit (QIAGEN, Germany). To analyze the taxonomic composition of the bacterial community, the 16S V4 universal bacterial primers (338F and 806R) were selected for the subsequent pyrosequencing. All PCR reactions were carried out with Phusion^®^ High-Fidelity PCR Master Mix (New England Biolabs). The TruSeq^®^ DNA PCR-Free Sample Preparation Kit (Illumina, United States) was used for library construction following manufacturer's recommendations and index codes were added. The library quality was assessed on the Qubit@ 2.0 Fluorometer (Thermo Scientific) and Agilent Bioanalyzer 2100 system. At last, the HiSeq2500 PE250 was used for on-machine sequencing and 250 bp paired-end reads were generated.

Paired-end reads were assigned to samples based on their unique barcode and truncated by cutting off the barcode and primer sequence and merged using FLASH (V1.2.7). Quality filtering on the raw tags was performed under specific filtering conditions to obtain high-quality clean tags using Quantitative Insights into Microbial Ecology software (QIIME, V1.9.1). In brief, (i) cut off the Raw Tags with continus low-quality values (threshold ≤ 19) reach a set length (threshold ≥ 3); (ii) filter out the Tags whose continuous high-quality base length is less than 75% of the length of Tags; (iii) remove the chimeric sequence through UCHIME Algorithm and Gold Database. Then, the filtered sequences were clustered into operational taxonomic units (OTUs) according to representative sequences using Uparse software (V7.0.1001) with a threshold of 97% sequence similarity and classified into the phylum, family, and genus levels against the Greengenes Database.

After resampling and normalization of the OTUs based on the minimum sequencing depth (29416), Alpha diversity was used to analyze the complexity of species diversity through five indices, including Shannon, Chao 1, Observed species, PD_whole_tree, and Simpson. Beta diversity was calculated after the OTU matrix was normalized using cumulative-sum scaling (CSS) by QIIME^51^, and graph was performed using R (V4.1.3) with the ‘*vegan*’ package. Linear discriminant analysis Effect Size (LEfSe) analysis^18^ was performed to explore microbial features of hypoxic and normoxic groups, and the LDA score was 3.

### Metabolomic profiling of rat feces and serum

Fecal pellets (60 mg) and serum (100 μL) were mixed with L-2-chlorophenyl alanine, C-17 and methanol to extract supernatant for liquid chromatography-mass spectrometry (LC–MS) analysis. An ACQUITY UHPLC system (Waters Corporation Milford, USA) coupled with an AB SCIEX Triple TOF 5600 System (AB SCIEX, Framingham, MA) was used to analyze the metabolic profiling in both Electrospray Ionization (ESI) positive and ESI negative ion modes. An ACQUITY UPLC BEH C18 column (1.7 μm, 2.1 × 100 mm) was employed in both positive and negative modes.

The acquired LC–MS raw data were analyzed by the Progenesis QI software (Waters Corporation Milford, USA) using the following parameters. The precursor tolerance was set at 5 ppm, fragment tolerance was set at 10 ppm, and retention time (RT) tolerance was set at 0.02 min. Internal standard detection parameters were deselected for peak RT alignment, isotopic peaks were excluded for analysis, noise elimination level was set at 10.00, and minimum intensity was set to 15% of base peak intensity. The resulting matrix was further reduced by removing any peaks with missing values (ion intensity = 0) in more than 60% samples. The internal standard was used for data quality control (reproducibility). The positive and negative data were combined to get combined data which was imported into SIMCA software package (V14.0, Umetrics, Umeå, Sweden). Principle component analysis (PCA) and (orthogonal) partial least squares discriminant analysis ((O)PLS-DA) were carried out to visualize the metabolic alterations among groups. The default 7-round cross-validation was applied with 1/seventh of the samples being excluded from the mathematical model in each round to guard against overfitting. The Hotelling’s T^2^ region, shown as an ellipse in score plots of the models, defines the 95% confidence interval of the modeled variation. Variable importance in the projection (VIP) ranks the overall contribution of each variable to the OPLS-DA model. The differential metabolites were selected based on a statistically significant threshold of VIP > 1 and *p*-value less than 0.05 from a two-tailed Student’s t-test on the normalized peak areas. Based on the KEGG database^52^, the differential metabolites were subjected to pathway enrichment analysis, and the differential metabolites were mapped to the KEGG database using the KEGG ID. *P-*value less than 0.05 is a significantly enriched pathway.

### Statistical analysis

Unpaired Student’s t-test for body weight, CRH, ACTH, CORT, TRH, TSH, tT_4_, tT_3_, fT_4_, and fT_3_, and Mann Whitney test for Shannon, Chao 1, Observed species, PD_whole_tree, and Simpson test was performed using Prism 7 software (GraphPad, San Diego, CA), and represented as mean ± standard error (SEM), with the significance criterion of *p* < 0.05 for two-tailed test.

The R packages “*stats*” and “*mediate*” were used for the regression analysis and CMA^53^. We examined whether fecal or serum metabolites (Mediator, M) mediate the causal mediate effect between intestinal bacteria and serum hormones. The variable included in causal mediation models needs to meet the four main assumptions using the generalized linear model (GLM). The four assumptions include (1) X can significantly predict Y (Y = β_0_ + β_1_X + e_1_), (2) X can significantly predict M (M = β_0_ + β_2_X + e_2_), (3) M will significantly predict Y (Y = β_0_ + β_3_M + β_4_X + e_3_) while adjusting X, (4) the relationship between X and Y was weakened, controlling for M. The treatment of hypoxia or normoxia was controlled as adjustment variables in GLM. We further estimated the average causal mediation effect (ACME, β_2_ × β_3_), average direct effect (ADE, β_4_), and total effect (β_1_) using *mediate* package. *P*-value less than 0.05 is a significant correlation.

## Supplementary Information

Supplementary Figures.

Supplementary Table 1.

## Data Availability

The sequence datasets generated during the current study are available in the SRA database under accession code PRJNA934696.
