# Supplementary material for: Association analysis of gut microbiota-metabolites-neuroendocrine changes in male rats acute exposure to simulated altitude of 5500 m
Source: Sci Rep. 2023 Jun 7;13:9225. doi: 10.1038/s41598-023-35573-y (PMC10247716; doi:10.1038/s41598-023-35573-y)
Supplement: Supplementary file 1 — Supplementary Figures. [file 41598_2023_35573_MOESM1_ESM.pdf]

Supplementary Figure 1

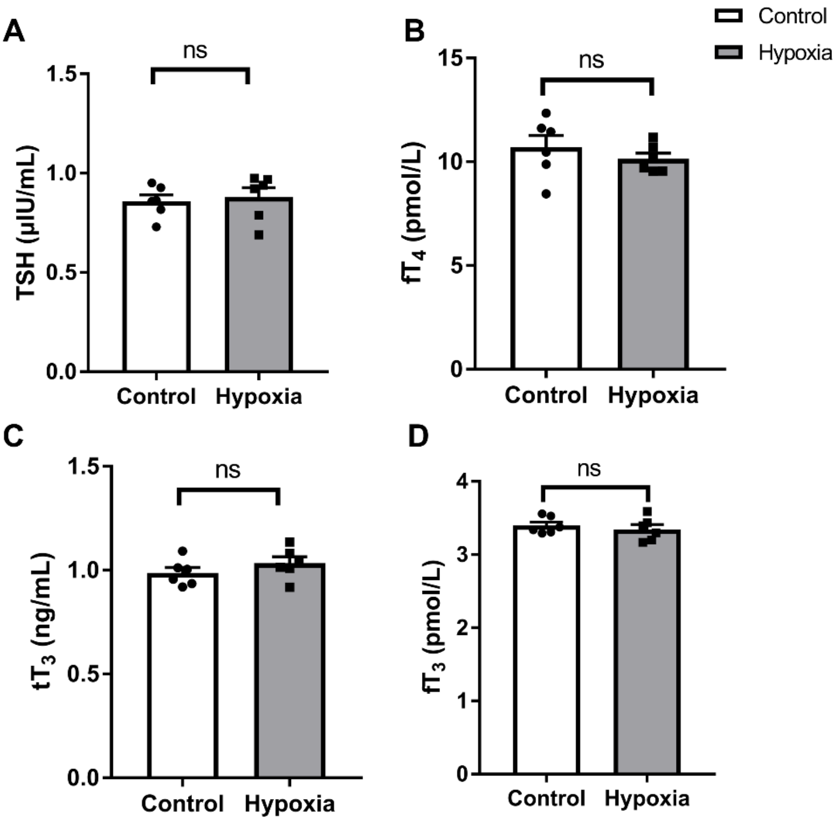

Supplementary Fig.1 Effect of simulated altitude at 5500m on serum levels of TSH (a), tT_3 (b), fT_4 (c), fT_3 (d). Data were expressed as mean ± SEM. ns. means $p>0.05$. n=6/group. TSH: thyroid-stimulating hormone; fT_4: free thyroxine; tT_3: triiodothyronine; fT_3: free triiodothyronine.

Supplementary Figure 2

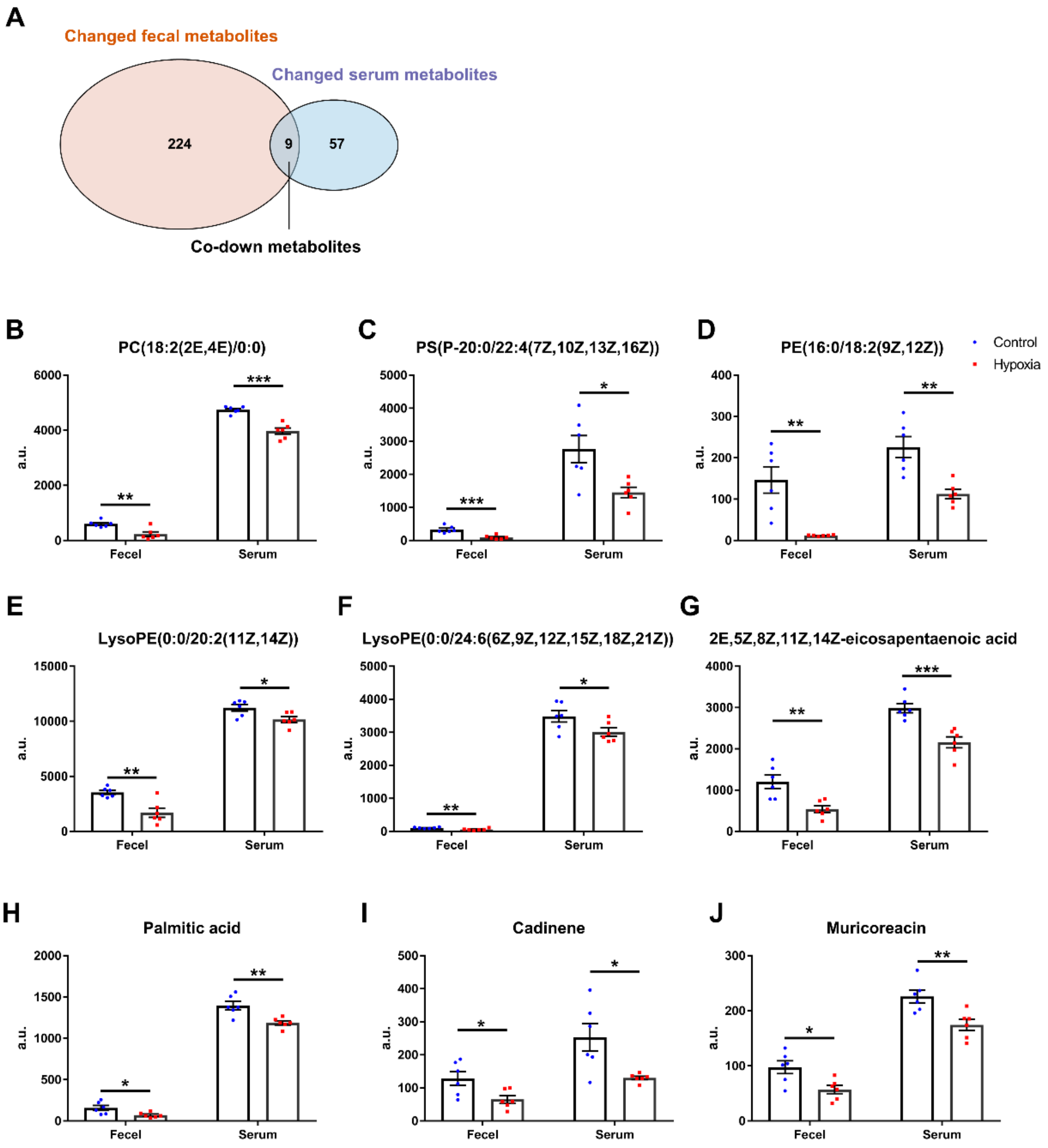

Supplementary Fig.2 Venn plot (a) of the changed metabolites in feces and serum. Bar plot showed the nine co-decreased metabolites in feces and serum (b-j). *p < 0.05,**p < 0.01, ***p < 0.001 vs. control group.
